# Sensitization to avian and fungal proteins in different work environments

**DOI:** 10.1186/s13223-023-00852-w

**Published:** 2023-11-13

**Authors:** Silvia Sánchez-Díez, Xavier Muñoz, Tomás Montalvo, Iñigo Ojanguren, Christian Romero-Mesones, Juan Carlos Senar, Victor Peracho-Tobeña, María-Jesús Cruz

**Affiliations:** 1 Servicio de Neumología, Departamento de Medicina, Hospital Universitario Vall d’Hebron, Universidad Autónoma de Barcelona, Passeig Vall d’Hebron, 119, 08035 Barcelona, Spain; 2 CIBER Enfermedades Respiratorias (Ciberes), Madrid, Spain; 3 Servicio de Vigilancia y Control de Plagas Urbanas Agencia de Salud Pública de Barcelona, Barcelona, Spain; 4 CIBER de Epidemiología y Salud Pública (Ciberesp), Madrid, Spain; 5 Departamento de Ecología Evolutiva y de la Conducta, Museo de Ciencias Naturales de Barcelona, Barcelona, Spain

**Keywords:** Hypersensitivity pneumonitis, IgG, Birds, Fungi, Exposed individuals

## Abstract

**Introduction:**

Hypersensitivity pneumonitis (HP) is usually caused by the inhalation of avian and fungal proteins. The present study assesses a cohort of Urban Pest Surveillance and Control Service (UPSCS) workers with high exposure to avian and fungal antigens, in order to identify their degree of sensitization and the potential risk of developing HP.

**Methods:**

Workers were divided according to their work activity into Nest pruners (Group 1) and Others (Group 2). All individuals underwent a medical interview, pulmonary function tests and the determination of specific IgG antibodies. Antigenic proteins of pigeon sera were analysed using two-dimensional immunoblotting. Proteins of interest were sequenced by liquid-chromatography–mass spectrometry (LC–MS).

**Results:**

101 workers were recruited (76 men, average age: 42 yrs); (Group 1 = 41, Group 2 = 60). Up to 30% of the study population exhibited increased levels of IgGs to pigeon, small parrot and parrot, and up to 60% showed high levels of Aspergillus and Penicillium IgGs. In Group 1, specific parakeet and Mucor IgGs were higher (*p* = 0.044 and 0.003 respectively) while DLCO/VA% were lower (*p* = 0.008) than in Group 2. Two-dimensional immunoblotting showed protein bands of 20–30 KDa recognized by HP patients but not by workers. LC–MS analysis identified Ig Lambda chain and Apolipoprotein A-I as candidate proteins for distinguishing HP patients from exposed workers.

**Conclusions:**

Two pigeon proteins were identified that may play a role in the development of pathological differences between HP patients and exposed workers. DLCO/VA may have a predictive value in the development of HP disease.

## Introduction

Hypersensitivity pneumonitis (HP) is an interstitial lung disease characterized by bronchoalveolar inflammation that occurs, in some genetically predisposed individuals, after the repeated inhalation of certain organic substances (e.g., avian and fungal proteins) [1, 2]. HP has recently been classified into two different forms: acute/inflammatory and chronic/fibrotic. The acute form is related to cellular inflammation and appears after discontinuous exposure to high antigen levels. Nevertheless, it often resolves with avoidance of the causative antigen. The chronic form is characterized by fibrotic areas inside the lungs that appear due to repetitive exposure to low antigen doses. It can cause respiratory insufficiency, which compromises patient survival and in some cases is irreversible [3, 4].

The diagnosis of HP remains challenging because of the absence of a gold standard. In fact, in clinical practice HP patients are diagnosed on the basis of a combination of clinical, imaging and laboratory findings. In this context, the presence of specific IgGs against a particular antigen in serum [5–8] or the specific inhalation challenge (SIC) are useful tools for the diagnosis of HP and for the identification of the causative antigen [9, 10].

In large cities, the structure of bird populations is not ecologically balanced, causing certain species to proliferate out of control and become pests [11]. They are monitored and controlled by municipal services, private pest control firms or research groups. Their staff work in activities with high exposure to birds and represent a possible risk group for the development of HP, given that avian population surveillance and control is one of their most important functions. To our knowledge, this is the first study carried out in different types of workers whose duties include the capture of birds such as pigeons and parrots, the destruction of nests, tree pruning, or the direct handling of birds for data collection, and so they are highly exposed to avian and fungal proteins during their workday. In these workers, the prevalence of sensitization to avian or fungal proteins is still unknown. In this sense, the specific proteins that cause HP pathology are still unknown, and may be of diagnostic value for discriminating between exposed but asymptomatic individuals and patients suffering from the disease. Several groups have identified antigenic substances found in bloom, serum, droppings [12] and/or intestinal mucin of different birds [13]. IGLL-1 and Proproteinase E (ProE) are examples of proteins recently identified as causative antigens of bird-related HP (BRHP) [14, 15] but many others remain to be discovered.

The present study assesses a cohort of Urban Pest Surveillance and Control Service (UPSCS) workers with high exposure to avian and fungal antigens, in order to determine their degree of sensitization to these antigens and the potential risk of developing HP. The comparison of this cohort with patients diagnosed with HP due to bird exposure may serve to identify antigenic proteins with diagnostic value.

## Material and methods

### Study population

We performed a prospective longitudinal study including bird researchers and/or managers at the Urban Pest Surveillance and Control Service (UPSCS) of the Barcelona Public Health Agency (ASPB), Municipal Institute of Parks and Gardens in Barcelona and employees of private urban pest control firms (n = 101). These individuals underwent a medical interview regarding their professional and domestic exposure to birds and fungi and pulmonary function tests to rule out respiratory symptoms. Blood extraction was performed in all subjects and after blood centrifugation (3000 rpm for 10 min) serum samples were obtained and stored at − 80 °C until analysis.

Patients with HP due to pigeon exposure (n = 5) who were diagnosed, according to the criteria proposed by Schuyler and Cornier [5], were included in the study as the control group for western blot analyses. The diagnosis was made when at least 4 major and 2 minor criteria were present and if other conditions with similar characteristics were ruled out. The major criteria were symptoms consistent with HP, evidence of appropriate antigen exposure in medical history and/or detection of specific precipitins in serum and/or bronchoalveolar lavage (BAL), findings consistent with HP on chest plain films or chest computed tomography, lymphocytosis in BAL (when performed), histological changes consistent with HP, and/or positive SIC. The minor criteria were bilateral basal crackles, decrease in DLCO, and/or arterial hypoxemia, at least after exercise.

### Antigen extract preparation

Blood from different types of birds (parakeet, small parrot and parrot) was collected and centrifuged to obtain serum samples. Commercially available pigeon serum (Rockland Immunochemicals Inc., Limerick, Ireland), *Aspergillus fumigatus, Penicillium frequentans* and *Mucor mucedo* extracts (Bial-Aristegui, Bilbao, Spain) were used. Protein concentration of avian serums was determined by the Bicinchoninic acid (BCA) method (Pierce Chemical Co., Rockford, USA).

### Specific IgG antibody determination

Specific IgG antibodies against avian and fungal proteins were determined in serum samples of all individuals of the study using a direct ELISA method.

Wells of high-binding microtiter plates (Costar, Cambridge, USA) were coated with avian serums and fungal extracts (2 µg protein/well) in coupling buffer (Na_2_CO_3_/NaHCO_3_, 0.2 M, pH 9.6) overnight at 4 °C. The wells were blocked (300 µL/well) with PBS/1% BSA for 1 h at 37 °C. After washing three times with PBST (PBS with 0.1% Tween-20), plates were incubated for 1 h at 37 °C with 200 µL/well of serum samples and negative and positive controls at 1/500 dilution in PBS/0.1% Gelatin/0.02% Tween-20. After three washes with PBST, plates were incubated for 1 h at 37 °C with 200 µL/well of mouse anti-human IgG-HRP antibody (SouthernBiotech, Birmingham, USA) at 1:1000 dilution in PBS/0.1% Gelatin/0.02% Tween-20. Wells were washed three times and the reaction was developed with 4.9% H_2_O_2_ and 3,3′,5,5′-tetramethylbenzidine in acetate buffer (pH 5.5) at 25 °C in the dark. After 20 min, the reaction was stopped with 2 M H_2_SO_4_ and read at 450 nm with a plate reader (Infinite M Nano, Tecan, Männedorf, Switzerland). Results were expressed as optical density (OD) and Magellan software (Tecan Austria GmbH, Grödig, Austria) was used for calculating the results.

Values above the mean plus 2 SDs of the results obtained in a control population of 30 healthy individuals previously studied in our laboratory were considered positive. The cut-offs were 0.284, 0.193, 0.294, 0.348, 0.508, 0.417, 0.258 and 0.687 OD_450nm_ for pigeon, small parrot, parrot, parakeet, duck, *Aspergillus fumigatus, Mucor mucedo and Penicillium frequentans*, respectively.

### Pulmonary function testing

A complete pulmonary function study including spirometry, static pulmonary volumes and diffusion capacity of the lung for carbon monoxide (DLCO) was carried out in all participants on a MasterLab system (MasterLab, Jaeger, Germany), in accordance with the guidelines of the European Respiratory Society and American Thoracic Society [16]. Static pulmonary volumes were measured using the plethysmography method and the diffusion study was carried out using the single-breath carbon monoxide method. The theoretical values were the ones proposed by the European Respiratory Society and American Thoracic Society [17, 18]. A restrictive ventilatory pattern was defined as forced vital capacity (FVC) < 85% of the predicted value with forced expiratory volume in 1 s (FEV_1_)/FVC ratio > 80% in the absence of a static lung volume study, together with a total lung capacity (TLC) < 80% of the predicted value, when these tests were performed. An obstructive ventilatory pattern was established on the basis of FEV1/FVC ratio < 70% together with FEV1 < 80% of the predicted value. The DLCO was considered decreased at values of < 80% of the predicted value.

### 1D/2D electrophoresis gel and western blot analysis

For 1D electrophoresis performance, sample buffer was previously prepared diluting 2-mercaptoethanol at 1/10 in Laemmli buffer 4x (Bio-Rad, Madrid, Spain). Avian serums (850 µg protein/1D gel for pigeon and small parrot serums) were then mixed with sample buffer at a 3:1 proportion. After heating the samples for 5 min at 90 °C in the water bath, samples were loaded onto preparative Criterion TGX Stain-Free gels 4–20% (Bio-Rad, Madrid, Spain) in accordance with the manufacturer’s manual. For 2D electrophoresis, avian serums (100 µg protein/strip) mixed with rehydration solution (pure H_2_O/8.5 M Urea/2% Chaps/0.5% IPG buffer/0.002% Bromophenol blue) were loaded onto immobilized pH gradient strips (IPG strip, pH 3–10, 7 cm, General Electric Healthcare, Boston, USA). Isoelectric focusing (IEF) was then carried out using the IPGphor IEF system (General Electric Healthcare, Boston, USA) and following the manufacturer’s instructions. IPG strips were then equilibrated and loaded onto Mini-PROTEAN TGX Stain-Free gels 4–20% (7 cm, IPG/prep, Bio-Rad, Madrid, Spain). After 1D or 2D electrophoresis, proteins were stained with Coomassie blue or blotted to a PVDF membrane using the Trans-Blot Turbo transfer system (Bio-Rad, Madrid, Spain). After blocking the membranes with nonfat dry milk (Bio-Rad, Madrid, Spain) for 1 h at RT and gentle shaking, washing steps were carried out with PBST (PBS with 0.05% Tween-20).

Western blots were performed by incubating serum samples diluted 1/100 in PBS for 2 h at RT with gentle shaking. After washing three times with PBST, membranes were incubated with goat anti-human IgG-HRP (SouthernBiotech, Birmingham, USA) diluted 1/2000 (for 1D blots) or 1/4000 (for 2D blots) in PBS for 1 h at RT. Membranes were then washed three times with PBST and incubated with the enzyme substrate for 5 min at RT (Kit Clarity Western ECL Substrate, Bio-Rad, Madrid, Spain), in accordance with the supplier’s recommendations. Image acquisition was carried out with the Odyssey Fc Imaging System and the Image Studio Lite software (LI-COR, Nebraska, USA). Spot analyses in 2D blots were performed using the PDQuest 2D software (Bio-Rad, Madrid, Spain). Healthy volunteers (HV; n = 3) without previous avian exposure and patients with BRHP due to pigeons (n = 5), who were diagnosed between 2009 and 2016 at our center (Hospital Universitari Vall d’Hebron, Barcelona, Spain) according to the criteria proposed by Schuyler and Cornier [5] were included as controls.

### Mass spectrometry analysis

Spots recognized by HP patients but not by workers nor HV were excised from 2D stained gels and digested with trypsin according to standard protocols. The tryptic peptides were analyzed with a maXis UHR-Qq-TOF mass spectrometer (Bruker Daltonics, Bremen, Germany) and subsequently sequenced using both the DeNovo and the Spider Homology Search tools from the PEAKS Studio 5.3 software (Bioinformatics Solutions Inc., Waterloo, ON, Canada).

### Statistical analysis

The normal distribution of the data was evaluated with the Shapiro–Wilk test. For categorical variables, data were analysed using chi-square or Fisher’s exact test and are expressed as absolute numbers and their corresponding percentages. For quantitative variables, data were analysed using Mann–Whitney *U*-test or Unpaired *T*-test, as appropriate, and data are shown as medians and ranges. Analyses were conducted using GraphPad Prism 6 for Windows (version 6.01, GraphPad Software Inc, San Diego, California, USA) and IBM SPSS Statistics (version 26, IBM Corporation, Armonk, New York, USA). Differences with a *p*-value < 0.05 (two-tailed) were considered to be significant.

## Results

### Characteristics of the study population

Demographic and clinical characteristics of the workers included in the study are shown in Table 1. The participants were divided according to their work activity into Nest pruners (Group 1, n = 28) and Others (Group 2, n = 73; biologists, n = 11; pest control staff, n = 28; others, n = 10). There was a significant difference between the median age of participants (Group 1: 49 yrs, 37–64; Group 2: 38 yrs, 20–62) (*p* < 0.0001). Workers in Group 1 had higher TLC values (% predicted) (Group 1 median, range 102, 73–140; Group 2 median, range 96, 74–158; *p* = 0.034) and lower DLCO/VA values (% predicted) (Group 1 median, range 80, 63–96; Group 2 median, range 88, 63–113; *p* = 0.008). No significant differences were observed between groups for other lung function parameters.

**Table 1:** Demographic, lung function and exposure data of the subjects in the study

	Nest pruners (n = 28)	Others (n = 73)	*p* value
Sociodemographic and clinical data			
Age (years), median (range)	49 (37–64)	38 (20–62)	< 0.0001
Sex (male), n (%)	23 (82)	53 (73)	0.441
Smoking habit, n (%)			0.299
Smoker	5 (18)	16 (22)	
Former smoker	9 (32)	17 (23)	
Non-smoker	8 (29)	36 (49)	
Pulmonary function test			
FVC (% predicted), median (range)	95 (70–122)	100 (72–127)	0.144
FEV1 (% predicted), median (range)	97 (70–137)	101 (74–144)	0.538
FEV1/FVC (% predicted), median (range)	78 (67–93)	82 (56–100)	0.101
TLC (% predicted), median (range)	102 (73–140)	96 (74–158)	0.034
DLCO/SB (% predicted), median (range)	82 (60–110)	87 (62–119)	0.449
DLCO/VA (% predicted), median (range)	80 (63–96)	88 (63–113)	0.008
Job exposure data			
Pruner, n (%)	28 (100)	0 (0)	< 0.0001
Gardener, n (%)	0 (0)	6 (8)	
Bird capture, n (%)	0 (0)	4 (6)	
Biologist (bird population control), n (%)	0 (0)	11 (15)	
Administrative worker, n (%)	0 (0)	10 (14)	
Pest control, n (%)	0 (0)	28 (38)	
Others, n (%)	0 (0)	10 (14)	
Domestic exposure data			
Feather duvet, n (%)	7 (25)	25 (34)	1.000
Birds, n (%)	3 (11)	11 (15)	1.000

Certain variables have missing values in some groups: Nest pruners (6 for smoking habit, 3 for all pulmonary function variables, 11 for feather duvet and 6 for domestic bird exposure); Others (4 for smoking habit, 1 for all pulmonary function variables, 4 for job exposure data, 8 for feather duvet and 4 for domestic bird exposure)

### Specific IgG levels to avian and fungal proteins

**Fig. 1:**
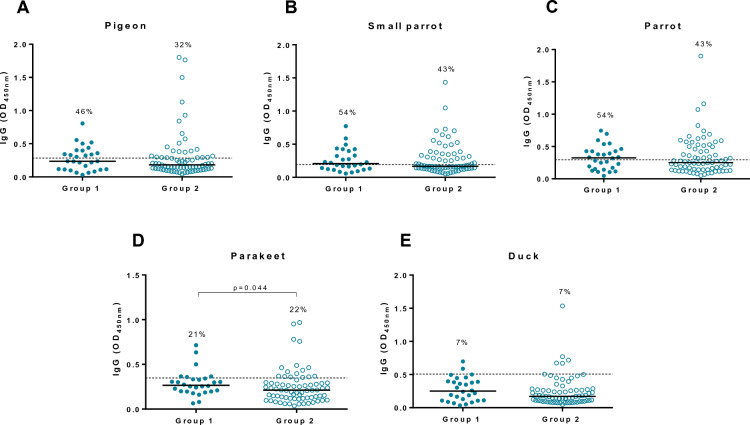
Specific IgG antibody levels against different birds in the study population**. A** Pigeon; **B** Small parrot; **C** Parrot; **D** Parakeet and **E** Duck. Dotted line in each graph indicates the cut-off previously established in our laboratory for each antigen using a cohort of 30 healthy subjects. The cut-offs for pigeon, small parrot, parrot, parakeet and duck were 0.284, 0.193, 0.294, 0.348 and 0.508 OD_450nm_ respectively. The percentage indicates the positivity of the group for each antigen. OD, optical density

**Fig. 2:**
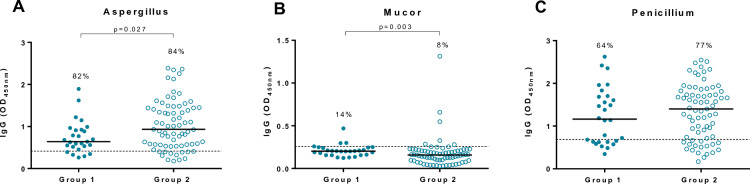
Specific IgG antibody levels against different fungi in the study population. **A** *Aspergillus fumigatus*; **B** *Mucor mucedo* and **C** *Penicillium frequentans*. Dotted line in each graph indicates the cut-off previously established in our laboratory for each antigen using a cohort of 30 healthy subjects. The cut-offs for *Aspergillus fumigatus, Mucor mucedo and Penicillium frequentans* were 0.417, 0.258 and 0.687 OD_450nm_ respectively. The percentage indicates the positivity of the group for each antigen. OD, optical density

Specific parakeet IgG levels were higher in Group 1 than in Group 2 (Group 1 OD median, range 0.266, 0.064–0.715; Group 2 OD median, range 0.212, 0.036–0.968; *p* = 0.044) (Fig. 1d). No significant differences were observed between groups for the other type of avian antigens. However, the percentages of workers with specific IgG levels above the established cut-offs in Group 1 were higher for pigeon, small parrot and parrot (46%, 54% and 54% respectively) compared to Group 2 (32%, 43% and 43% respectively) (Fig. 1a–c respectively). Regarding fungal exposure, workers in Group 2 exhibited increased levels of specific aspergillus IgGs (Group 2 OD median, range 0.934, 0.18–2.387; Group 1 OD median, range 0.642, 0.263–1.894; *p* = 0.027) (Fig. 2a) while specific mucor antibodies were higher in Group 1 (Group 1 OD median, range 0.203, 0.123–0.47; *p* = 0.003; Group 2 OD median, range 0.158, 0.028–1.313) (Fig. 2b).

### Protein recognition pattern in workers and HP patients

**Fig. 3:**
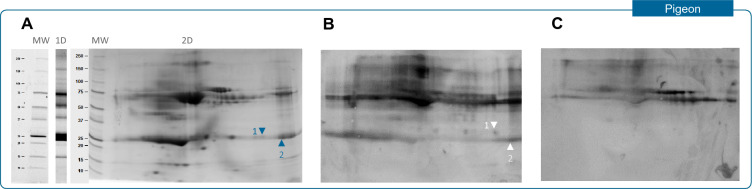
Protein composition of pigeon serum by one and two dimensional electrophoresis (**A**) and two dimensional immunoblots of a patient with HP due to pigeon exposure (**B**) and a worker in the study with high exposure to pigeon (**C**)

One-dimensional and 2D electrophoresis of pigeon serum showed different protein bands and spots of interest respectively, mostly in the molecular weight range of 20–25 KDa and 50–75 KDa (Fig. 3a). A differential pigeon protein recognition pattern was observed in the low molecular weight range (20–25 KDa) when comparing two-dimensional immunoblots between patients suffering from HP due to pigeon exposure (Fig. 3b) and workers in the study population exposed to this type of bird (Fig. 3c). After sequencing, two pigeon proteins recognized by all BRHP patients but not by workers were identified (Ig Lambda chain and Apolipoprotein A-I, Table 2) (Accession numbers in UniProt database: A0A2I0LZC1, A0A2I0LQE2, A0A2I0LQE2).

**Table 2:** Identification of pigeon serum proteins by Liquid chromatography–mass spectrometry

Spot ID^a^	MW (kDa)^b^	Protein identified (theoretical MW in kDa)	Accession Number^c^	Score	Peptides	Sequence coverage (%)^d^
1	25	Ig Lambda chain (22.9)	A0A2I0LZC1	136.7	3	20.6
1	25	Apolipoprotein A-I (30.6)	A0A2I0LQE2	169.2	8	27.3
2	25	Apolipoprotein A-I (30.6)	A0A2I0LQE2	524.9	18	52.3

*MW* molecular weight

^a^Each spot is described by its number on the 2D gel and WB (Fig. 3a, b)

^b^Apparent MW extrapolate from the standard in gel

^c^Accession number in UniProt database

^d^Percentage of the protein covered by matched peptides

**Fig. 4:**
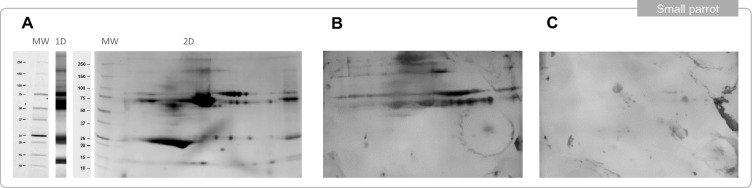
Protein composition of small parrot serum by one and two dimensional electrophoresis (**A**) and two dimensional immunoblots of two workers in the study with high exposure to small parrot (**B**, **C**)

One-dimensional and 2D electrophoresis of small parrot serum showed a similar protein pattern to that observed for pigeon serum with proteins of high and low molecular weight range (Fig. 4a). Two-dimensional western blots from different workers exposed to small parrot presented different recognition profiles: one of them recognized a great many small parrot proteins, mostly in the high weight molecular range (75–250 KDa) (Fig. 4b) while the other only recognized a few proteins (Fig. 4c). After looking at the clinical history, the most sensitized worker had been diagnosed with sarcoidosis 15 years previously and presented crackles during the clinical examination.

## Discussion

The present study assesses the degree of sensitization to avian and fungal antigens and the potential risk of developing HP in a cohort of researchers, pest control workers and parks and garden staff. The results demonstrate that all individuals included in the study had a high degree of sensitization to avian and fungal proteins. In particular, workers involved in nest pruning had increased levels of specific parakeet and Mucor sp IgGs and lower DLCO/VA values while other types of workers (e.g., those employed in urban pest control, bird population control or administration) showed high levels of specific IgGs against Aspergillus sp. In addition, in this study Ig Lambda chain and Apolipoprotein A-I were identified as candidate proteins for distinguishing HP patients from workers exposed to pigeon.

Increased serum levels of specific IgG antibodies confirm the existence of sensitization to a specific antigen after a previous exposure. Epidemiological studies have documented a relationship between exposure to organic antigens and the level of serum-specific IgG antibodies against these antigens [19] and/or the prevalence of HP [20–22]. According to our results, workers employed in nests pruning presented high levels of specific IgG to avian and fungal antigens. Similarly, in a study of 41 patients suffering from HP, Miyagawa et al. [23] concluded that sera from all patients had high titers of antibodies against a specific fungus. Rodrigo et al. [24] also demonstrated that patients who developed pigeon breeder’s disease synthesized high levels of IgG antibodies against pigeon proteins. However, IgG determination is merely evidence of antigenic sensitization, and exposed but asymptomatic individuals can also present high IgG levels against a specific antigen without having the disease [25–27]. In fact, there are studies demonstrating that up to 50% of healthy individuals exposed to birds can be sensitized to avian antigens [24]. Erkinjuntti-Pekkanen et al. [28] concluded that not only farmer’s lung patients but also between 30 and 60% of control farmers developed specific antibodies against the different microbes tested. In fact, workers in our study employed in activities other than nest pruning also had increased levels of specific IgGs against birds and fungi.

The major limitations of specific IgG measurement are the unavailability of validated antigen preparations for most agents that cause HP, and the fact that they are a marker of exposure but not of disease. Recently, several groups have reported certain antigenic substances that are found in bloom, serum, droppings and/or intestinal mucin of different birds that might be used as diagnostic markers of the disease [12–15]. Using a quantitative automated fluorimetric enzyme immunoassay, Koschel et al. [12] defined a threshold value for discriminating healthy subjects from patients with bird fancier’s lung for antigen-specific IgG antibodies against goose and duck feathers. Shirai et al. [14] found that serum IgG antibodies against immunoglobulin lambda-like polypeptide-1 (IGLL-1) contained in pigeon serum were increased in patients with bird-related HP compared to control subjects. Rouzet et al. [15], identified this IGLL1 and ProE in bloom and droppings of pigeons as biomarkers of disease when comparing sera from patients with bird fancier’s lung, asymptomatic exposed controls, and healthy volunteers. Our study also identified Ig Lambda chain (22.9 KDa) and Apolipoprotein A-I (30.6 KDa) as candidate proteins for distinguishing HP patients from workers exposed to pigeon.

In the present study workers involved in nest pruning had low DLCO/VA values. According to the literature, decreased DLCO/VA values may reflect qualitative impairment of the alveolar-capillary membrane and can be associated with different abnormal conditions; diffuse alveolar-capillary disease, reduced alveolar surface or capillary density, or diminished hemoglobin levels [29, 30]. The individuals in the nest pruning group had the highest specific IgG levels and, in view of their job description, probably had the highest exposure to avian and fungal antigens. This high exposure could be the reason for the differences observed in DLCO/VA values compared to the group who performed other activities with lower antigenic exposure. In addition, low DLCO/VA values could predict DLCO reduction over time and disease progression [29]. In fact, in patients with HP the most frequent finding in lung function tests is a restrictive ventilatory disorder or an alteration in gas exchange, consisting of a decrease in DLCO values [31]. Moreover, we cannot rule out the influence of the older age of workers involved in nest pruning on the DLCO/VA values.

In this sense, a limitation of this article is that a follow-up study of workers with low DLCO/VA values has not been carried out. Although no cases of HP were diagnosed in the individuals in the nest pruning group, these lowered levels of DLCO/VA and the increased levels of specific IgG may indicate a greater predisposition to the development of disease in these workers. Therefore, further follow-up studies should assess the prognostic value of DLCO/VA as biomarker in the progression of HP. On the other hand, we cannot rule out that these workers may have specific IgG antibodies to other substances present in the environment. In this sense, the major limitation of specific IgG measurement is the unavailability of validated antigen preparations for most agents causing HP.

In conclusion, a high degree of sensitization to avian and fungal antigens was observed in the study population. In addition, we identified two pigeon proteins that may play a role in the development of pathological differences between HP patients and exposed workers; Ig Lambda chain and Apolipoprotein A-I. With these results, there is a need for health risk assessment and occupational risk prevention systems to work together to reduce exposure and associated risks.

## Data Availability

All data generated or analysed during this study are included in this published article (and its supplementary information files). For the identification of pigeon serum proteins by Liquid chromatography–mass spectrometry: Accession numbers in UniProt database: A0A2I0LZC1, A0A2I0LQE2, A0A2I0LQE2.
